# Medical Treatment Behaviour of the Elderly Population in Shanghai: Group Features and Influencing Factor Analysis

**DOI:** 10.3390/ijerph18084108

**Published:** 2021-04-13

**Authors:** Shangguang Yang, Danyang Wang, Chen Li, Chunlan Wang, Mark Wang

**Affiliations:** 1 School of Business, East China University of Science and Technology, 130 Meilong Road, Shanghai 200237, China; sgyang@ecust.edu.cn; 2 Institute of Future Cities, Department of Geography and Resource Management, The Chinese University of Hong Kong, Hong Kong 999077, China; chen.li@cuhk.edu.hk; 3 Chinese Modern City Research Center, School of Social Development, East China Normal University, Shanghai 200062, China; clwang@soci.ecnu.edu.cn; 4 School of Geography, The University of Melbourne, Parkville, VIC 3010, Australia; myw@unimelb.edu.au

**Keywords:** elderly population, medical treatment behaviour, influencing factors, Shanghai, China

## Abstract

Background: While Chinese cities are pursuing economic development, meeting citizen demand for medical treatment has only gradually been put on the agenda. Theoretically, in the second half of a person’s life, demand for medical treatment will rise sharply. Given limited medical resources, the match between demand and supply becomes more difficult. We conducted questionnaires in Shanghai to describe whether there are obvious group differences in the elderly population’s medical treatment options and provide empirical evidence on the determinants. Method: We collected 439 Shanghai Elderly Medical Demand Characteristics Questionnaires, which included five parts: personal information, health status, elderly person’s medical preference and expectation, satisfaction level for hospitals services, and medical insurance. We set up virtual explanatory variables according to the different medical behaviours of the elderly, and control variables composed of individual characteristics, socioeconomic characteristics, medical needs, medical resource availability, and medical expenditure. We used the MLR model to investigate medical treatment behaviour choice. Results: The medical treatment behaviour of the elderly population in Shanghai is affected by multiple factors. When experiencing physical discomfort, most of them choose to go to the hospital (64.69%). Age, income, household registration, and medical insurance reimbursement policy play a role in their decision-making. For general diseases, the proportion choosing specialist hospitals or community clinics is the highest (40.78%). Age, marital status, residential status, physical state, objective distance, medical expenses, and other factors have a significant impact. For severe diseases, they are more inclined (71.07%) to visit general hospitals, with the individual’s physical condition, living status, and accessibility to hospital resources more likely to affect their behaviour. Conclusion: Firstly, the importance of each factor varies depending on the conditions. Secondly, it may be more appropriate for China’s elderly health insurance system to set reimbursement rates based on the patient’s condition and disease type. Thirdly, medical behaviour has a distance friction effect, but the allocation of public service resources shows a strong centripetal concentration. It is necessary for the government to show due care about the regional distribution of the elderly population and to promote the rational distribution of medical resources in Shanghai.

## 1. Introduction

Population aging has become an irreversible development trend in many countries. The health and medical care problems faced by the elderly population have become increasingly prominent. Due to differences in the groups of elderly people, medical systems, and social welfare, the incompatibility of the medical supply and demand of the elderly is very prominent [1,2]. Now, politics and academia are very concerned about accurately understanding the growing medical needs of the elderly population and their medical behaviour [3,4,5].

Compared with Japan’s “rich frontier while old” and Singapore’s “rich first and then old”, China could be called “older before rich”. China is one of the first countries in the world where the combination of ageing challenges and economic development conditions emerged [6]. Shanghai is China’s most aged city. In 2017, the proportion of permanent residents over 65 years old reached 14.3% of Shanghai’s total permanent residents [7]. The increase in the proportion of the elderly has greatly increased social pension and medical expenditure and has also changed the labour supply pattern. Due to the gradual deterioration of their physiological function and the continuous decline of body resistance, the incidence rate is higher than other groups and has led to great demand for medical services. Theoretically, in the second half of a person’s life, their demand for medical treatment will rise sharply. When medical resources are limited and the supply has a certain time lag (e.g., the training required to work in a hospital for doctors in China is generally 8 years or even longer), this mismatch between demand and supply has become more evident. The health status of the elderly is an important reference point for individuals to seek medical treatment and determine their medical treatment choices. An important challenge for a comprehensive public health policy in response to an aging population lies in the diversity of the health and functional status of the elderly given that their physiological change may not match their age [3].

The number of medical visits and hospitalisations of the elderly are highly discrete. Western economists prefer to use micro-questionnaires. For demographic characteristics (race, gender, marital status, and education), economic factors (family income, employment status), the number of chronic diseases, public medical insurance, and commercial medical insurance are used to discuss the group differences in the medical service needs of the elderly [8]. Gender, age, and habits, among others will affect individual medical expenditure [9]. Smoking or exercise habits are some of the reasons for the difference in medical expenditure [10]. Marital status and residential arrangements will also directly affect their health status. The spouse or children ensure the timeliness and convenience of the elderly obtaining the medical care resources that they need [11]. Good education can increase their understanding of the disease, which facilitates prevention measures and access to treatment resources and information. Additionally, they can participate in disease diagnosis and treatment in a timely and effective manner [12].

At the same time, scholars also focus on the economic analysis of the efficiency and welfare of resource allocation in the medical service market [13]. The UK’s NHS plan recognises that unhealthy complex causes are rooted in personal lifestyle, community, and economic problems involved as well as the chaos, unfairness, and lack of responsibility for people’s needs shown by past medical care systems. Those over 60 who pay private health insurance premiums receive tax relief policies. Germany began to introduce long-term care insurance in 1995, which helped to improve at least two aspects of daily life activities, including hygiene, diet, mobility, and housework, that require regular or substantive assistance. In 2009, the eligibility for this in Germany was 11.5% for those over 65. Not only has it alleviated the government’s financial pressure, but it has also allowed most elderly people to support themselves when it comes to medical care. Singapore has the “Health Insurance Plan” and the “Value-Added Health Insurance Plan”, which are used to solve the medical expenses of insured persons for serious or chronic diseases. At the same time, in order to supplement the insufficiency of the health care financing system, a new low-cost insurance project called “Elder Shield” was implemented in June 2002 to provide economic protection for people suffering from severe disabilities. The United States pays attention to the comprehensive management of elderly patients. Geriatric hospitals are connected with the long-term care system to form a hierarchical medical care system. At the same time, it pays more attention to the important role of community care for the health of the elderly [14,15,16,17]. The maintenance and improvement of the health of the elderly involves the social system and socioeconomic status. The social system determines the distribution of social resources and existing lifestyle. An effective medical insurance system can increase the possibility of meeting individual medical service needs [18]. However, under the same system, the medical expenditure behaviour of the elderly has certain group characteristics [19]. Williams (1990) concluded that the socioeconomic status of the elderly is the most decisive factor affecting individual health status [20] and this relationship proved to be robust and continuous [21]. For example, in the United States, if low-income, uninsured or racial/ethnic minorities, immigrants, or rural elderly populations have poor access to quality oral care, their oral health is more likely to be poor [22]. The limited financial compensation provided by local governments in China, coupled with problems, such as unclear function positioning and low supply efficiency in public hospitals, have also led to social welfare losses [23,24].

With China’s growing aging population, the elderly population has become the group that has the largest demand for medical services. Their health needs and medical care have become the focus of society [25]. However, the distribution of medical facilities and resources is mainly driven by population and economic development, concentrated in Beijing, Shanghai, and other eastern coastal provinces. For some underdeveloped areas in the west, the current medical conditions cannot meet their needs [26]. In the mega city Shanghai, reforms and innovations in economic, social, cultural, and ecological aspects have been optimised and the urban spatial structure reconstructed. The miniaturisation and simplification of family size has directly weakened the family’s function of caring for the elderly [27]. China’s administrative system allocates personnel, money, and goods to a variety of public hospitals [28]. The referral reform has improved the overall accessibility of Shanghai’s public hospitals, but at the same time, it has exacerbated the inequality of access to medical resources in cities and communities [29]. Residents in relatively good physical condition choose social health service centres for medical treatment, while residents in poor physical condition choose general hospitals for medical treatment. However, the time cost of medical services (traffic time, waiting time) will significantly affect the individual’s medical decision [30,31]. Therefore, the allocation of medical resources will highly influence the behaviour of the elderly in seeking medical treatment.

There are three significant gaps in the previous literature. First of all, previous studies used a large-scale national survey database, which is more suitable for national research, but it does not represent problems faced in megacities of developing countries. Second, the behaviour of those seeking medical treatment is based on an individual’s choice under the combined effect of micro and macro factors, and the classification of elderly characteristics is not detailed enough. Third, studies on the elderly population’s medical treatment behaviour are rare and ignore an important factor: the severity of their condition.

Based on the analysis of first-hand questionnaire data, this study explored the differences in medical treatment behaviour between a variety of determinants using three conditions of the elderly population in Shanghai: physical discomfort, general disease, and severe disease (severe diseases generally include: malignant tumours, severe cardiovascular and cerebrovascular diseases, acute myocardial choking, stroke sequelae, major organ transplantation or hematopoietic stem cell transplantation, coronary artery bypass grafting, chronic liver function failure and decompensation period, injuries that may cause life-long disabilities, advanced chronic diseases, deep coma, permanent paralysis, severe brain injury, severe Parkinson’s disease, and severe mental illness). This study could provide a scientific basis for the Chinese megacity governments to formulate comprehensive medical reform measures.

## 2. Method

### 2.1. Study Population: Shanghai Elderly Medical Demand Characteristics Questionnaire

We used both qualitative and quantitative methods. For qualitative analysis, we analysed the empirical results of Shanghai’s medical policy measures and the individual characteristics of the elderly population (see Supplementary Materials). For quantitative analysis, we collected the first-hand data from the “Shanghai elderly medical demand characteristics questionnaire” from November 2017 to December 2017, and the second-hand data from statistical data, including government development reports of Shanghai Municipal Peoples Government and Shanghai Bureau of Statistics.

The questionnaire covered the following:

- Personal information in order to understand basic demographic characteristics (gender, age, marital status, and relationship);
- *Hukou* (the Hukou system is one of the major tools of social control employed by the state. The Chinese system was designed not merely to provide population statistics and identify personal status, but also to directly regulate the population distribution and serve many other important objectives desired by the state. If a person holds Shanghai hukou, it means he is a Shanghai native and enjoys institutional benefits within the Shanghainese jurisdiction. In terms of medical insurance, the gap between local hukou and non-local hukou in enjoying medical services is even more obvious. Shanghai clearly stipulates that the non-local hukou population cannot hold the medical insurance card for urban residents in Shanghai and therefore, they cannot enjoy Shanghai’s basic medical security rights) place and residential place;
- Socioeconomic status (education level, employment, and income);
- Health status in order to understand the reason for the medical service (self-evaluation of physical health and whether it involves chronic disease (common chronic diseases are mainly cardiovascular and cerebrovascular diseases, cancer, diabetes, chronic respiratory diseases, among which cardiovascular and cerebrovascular diseases include hypertension, stroke, and coronary heart disease);
- Medical preference and expectation in order to understand medical habits and behavioural choices;
- Satisfaction of hospitals and medical services in order to understand the current situation of the hospital and medical service supply; and
- Medical insurance mode and the level of success in using it in order to understand medical security status (whether basic medical insurance is held).

Through multi-stage stratified random sampling, we randomly selected:

(1) A number of districts in the city’s central area, suburbs, and outer suburban areas; then
(2) A number of streets in each district; and then
(3) Neighbourhood committees in each street.

The survey respondents were Shanghai residents aged 60 years and above. Before the formal survey, we conducted a small-scale pilot survey in three districts to ensure that the questionnaire design was reasonable. The formal survey was based on modifications of the initial one and it covered 12 districts in Shanghai. The survey covered 39 streets, 132 neighbourhood committees, and 6 rural communities. The respondents covered the areas divided by Shanghai’s inner ring, middle ring, and outer ring (these districts include Jing’an, Huangpu, and Hongkou in the urban core areas; Xuhui, Changning, Putuo, and Yangpu in the urban fringes; Pudong, Minhang, Baoshan, and Jiading in the suburbs; and Jinshan, Songjiang, Fengxian, Qingpu, and Chongming in the outer suburban areas). We obtained 439 valid questionnaires. Incomplete questionnaires, such as surveys with less than an entire section completed, with fewer than half the total items completed, or those with identical responses to every item, were excluded.

### 2.2. Dependent Variable

This study investigated medical needs and behaviour exhibited in three conditions: physical discomfort, general disease, and severe disease. When there is physical discomfort, elderly people have four choices: going to the hospital, going to the pharmacy, ‘endurance’ (stay at home), and taking other measures. Going to the hospital was set as the control group in this study because it is a direct medical treatment behaviour indicating that elderly people have higher health awareness and more sensitive and prudent medical judgement. Hospitals in Shanghai were classified according to a 3-tier system that represents a hospital’s ability to provide medical care, medical education, and conduct medical research. Hospitals were labelled as primary, secondary, or tertiary institutions. A primary hospital is typically a township hospital that contains less than 100 beds. They are tasked with providing preventative care, minimal health care, and rehabilitation services. Secondary hospitals tend to be affiliated with a medium size county or district and contain more than 100 beds but less than 500. They are responsible for providing comprehensive health services as well as medical education and conducting research on a regional basis. Tertiary hospitals round up the list as comprehensive or general hospitals at a city, provincial, or national level with a bed capacity exceeding 500. They are capable of providing specialist health services and medical education as well as scientific research, and thus they serve as medical hubs providing care to multiple regions. For general and severe diseases, the elderly population’s treatment options include going to a general hospital (tertiary hospitals), district hospital (secondary hospitals) and other specialised hospitals, or community clinics (primary hospital). Generally, elderly people will have access to the highest quality of medical supply when they choose a general hospital. Therefore, this study set going to a general hospital as the control group.

### 2.3. Independent Variables

Independent variables were composed of four groups: socioeconomic characteristics (gender, age, income, marriage status, cohabitation status, education level, rural or urban, and *hukou*), medical needs (health condition, chronic disease), medical resource availability (the level (the level refers to the nearest hospital level to one’s home, which is the primary, secondary, or tertiary institutions, mentioned above) and the distance of the nearest hospital from home from a subjective basis, the level and distance of the nearest hospital from home from an objective basis), and medical expenditure (understanding medical insurance reimbursement policy, preferring medical institutions with higher reimbursement rate).

### 2.4. Modelling and Estimation Methods

**Figure 1:**
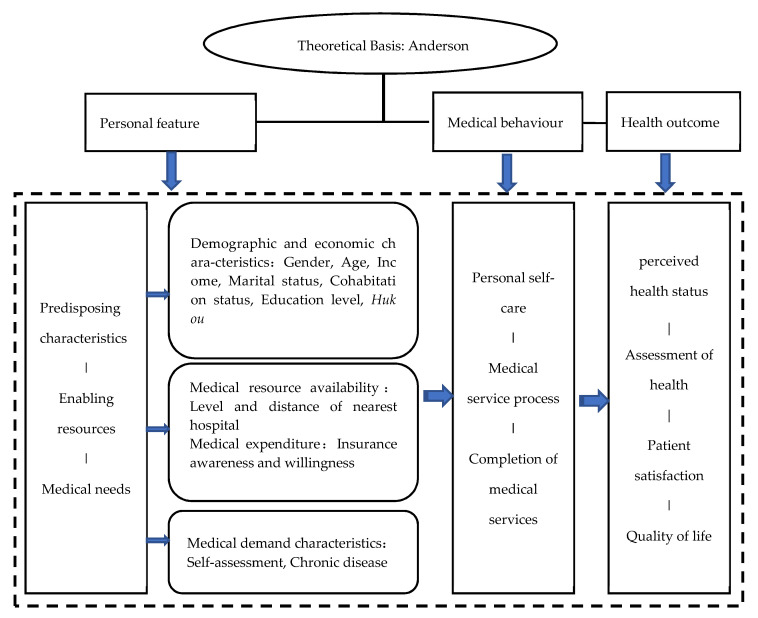
Theory of Medical Behaviour of Elderly People in Shanghai (in the Anderson model, the indicators under the “contextual characteristics” dimension are all based on “community/society” as the analysis unit, which is not applicable to this study, so it is omitted in Figure 1).

American medical sociologist Andersen created “The Behavioral Model of Health Service Use” in 1968 to analyse the influencing factors of medical and health use behaviour. The model takes into account the completeness of the theory and the feasibility of empirical evidence. It can comprehensively and systematically select measurement indicators and put forward research hypotheses. The latest version of the Anderson model was revised and completed in 2013, including four dimensions of “contextual characteristics”, “individual characteristics”, “health behaviours”, and “medical outcome outcomes”. It is a mature and concise model, including a one-way path relationship, two-way path relationship, intermediary relationship, and four types of parallel. The “individual characteristics” is the basic component of the Andersen model. It reflects the influence of the individual’s subjective and objective conditions on his medical behaviour, including three secondary indicators: “predisposing characteristics”, “enabling resources”, and “needs”. “Predisposing characteristics” is defined as containing demographic, genetics, social, and beliefs. “Enabling resources” means the individual capacity to access health services and availability of resources for health services. “Needs” means individual perceived medical needs. Though there are differences in the cultural background and the national medical and health system, due to China’s continuous opening up and its gradual integration with international standards, we need to apply the Anderson model to the empirical research in the field of medical and health in China with proper selection of measurement indicators. It would be helpful to accelerate the level of medical market opening in China. We collected the key elements that fit the definition of indicators of the classic Anderson model in the questionnaire. The demographic and economic characteristics were classified as predisposing characteristics, and medical resource availability and medical expenditure were classified as enabling resources (see Figure 1).

**Figure 2:**
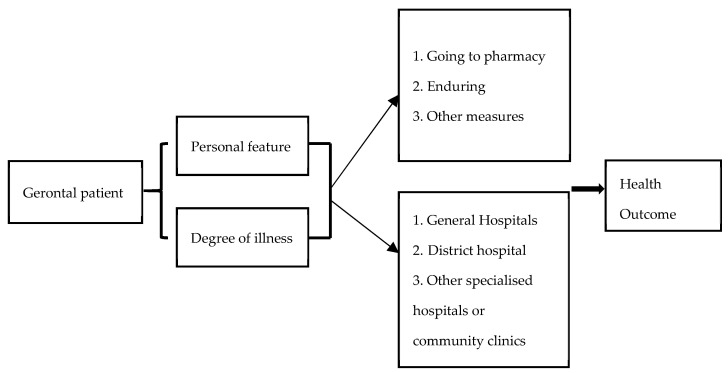
Selection of Gerontal patient.

At the same time, this study considered that under the control of the Chinese government, medical insurance and the household registration system leads to different medical costs and time spent by the elderly, which may interfere with whether and where to seek medical treatment for the elderly. After the elderly get sick, their behaviour is affected by individual characteristics and group characteristics. Health results will have a positive effect, but the cost of seeing a doctor is obviously a negative effect. Based on the measurement of costs and benefits, the elderly will make two decisions about going to see a doctor or no behavioural choice (see Figure 2). Therefore, empirical analysis of three different situations of slight discomfort, general disease, and serious disease was carried out in the empirical evidence, so as to distinguish the true effect of each influencing factor under different conditions.

In order to measure whether these factors influence the elderly population’s different medical needs and behaviour in Shanghai, we constructed the utility function representing individual medical health demand behaviour based on the medical health models established by many scholars [32,33,34,35]. Their modelling paradigm is from a micro individual utility theory perspective and obtains empirical evidence by building individual medical health demand models and using parameter estimation. The significance of their models lies in explaining individual medical treatment choices from the utility theory perspective, using quantitative methods to incorporate patients’ medical treatment choices into the unified model, and comes from a micro-perspective to discuss different factors that influence patients’ medical treatment behaviour and the quantitative relationship between these factors and such behaviour.

The utility model in this study is as follows:

(1) $$U_{ij}=U_{ij}\left(X_{i},N_{i},D_{i},E_{i}\right)\left(i=1,2,\cdots ,n;j=1,2,\cdots ,J\right)$$

In Equation (1), $U_{ij}\;$represents the random utility brought about by medical treatment behaviour j, which is an elderly individual i; $X_{i},N_{i},D_{i},\mathrm{and}\;E_{i}$ are four types of factors, respectively representing the elderly population’s socioeconomic characteristics, medical needs, medical resources availability, and medical expenditure, respectively.

The econometric model is obtained by processing the utility function:

(2) $$U_{ij}=\beta_{j}\;\cdot \;S_{i}+\varepsilon_{ij}\left(i=1,2,\cdots ,n;j=1,2,\cdots ,m\right)$$

In Equation (2), $S_{i}$ is the elderly individual i’s characteristic vectors, including $X_{i},N_{i},D_{i},\;E_{i}$, which varies with the individuals; $\varepsilon_{ij}$ is the random error term; and $\beta_{j}$ is the parameter to estimate.

When an elderly individual i shows medical treatment behaviour j, if and only if the medical health treatment has the greatest effect, the possibility that the elderly individual i takes medical treatment behaviour j is:

(3) $$\begin{array}{ll} P_{ij} & =P\left(U_{ij}>U_{ik},\forall k\neq j\right)\\ & =P\left(U_{ik}-U_{ij}\leq 0,\forall k\neq j\right)\\ & =P\left(\varepsilon_{ik}-\varepsilon_{ij}\leq \beta_{k}\;\cdot \;S_{i}-\beta_{j}\;\cdot \;S_{i},\forall k\neq j\right) \end{array}$$

Assuming $\varepsilon_{ij}$ conforms to the IIA condition and complies with the i-type extreme value distribution, it can then deduce that the possibility that the elderly individual i takes medical treatment behaviour j is:

(4) $$P_{ij}=\exp\left(\beta_{j}\;\cdot \;S_{i}\right)/\overset{m}{\underset{k=1}{\sum }}\exp\left(\beta_{k}\;\cdot \;S_{i}\right)$$

Equation (4) is the multiple value selection model. It can use maximum likelihood estimation (MLE) to obtain the estimated coefficient β. The log-likelihood function is:

(5) $$lnL_{i}\left(\beta_{1},\beta_{2},\cdots ,\beta_{m}\right)=\overset{m}{\underset{j=1}{\sum }}U_{ij}lnP_{ij}$$

If the elderly individual i chooses medical treatment behaviour j, then $U_{ij}$ is 1, and 0 otherwise. We chose multinomial logistic regression to predict our model

## 3. Results

### 3.1. Characteristics and Options

Table 1 and Appendix A show the differences in medical treatment behaviour of the elderly when they are in physical discomfort, suffering from general disease and severe disease. They show that when the elderly experience physical discomfort, most will choose to go to the hospital (64.69%), followed by the pharmacy (25.28%), enduring at home (6.83%), and only a small proportion take other measures (3.19%). When the elderly suffer from general disease, the proportion choosing specialised hospitals or community clinics is the highest, at 40.78%, followed by the district hospital (37.13%) and general hospital (22.10%). When suffering from severe disease, the elderly are more inclined to seek medical treatment in high-level hospitals, with the proportion as high as 71.07%.

### 3.2. The Influencing Factors of Medical Treatment Choices for Physical Discomfort

Whether elderly people go to the hospital is influenced by a variety of factors when it comes to physical discomfort. As shown in Table 2, some factors, such as demographic and economic characteristics, medical demand characteristics, medical resource availability, and medical expenditure, significantly influence the elderly people’s medical treatment choices.

First, in terms of socioeconomic characteristics, compared with people aged 60–64, the 70–74 group may be less willing to go to the pharmacy (OR = 0.46, 95%CI: 0.20, 1.06). Those who are 80 years old and above also have this pattern (OR = 0.27, 95%CI: 0.09, 0.88). Respondents were randomly selected for interviews. One interviewee said that the reason he went to the hospital was that “when I was young, the work intensity was heavy and exhausting. That resulted in physical problems when I get old. I was the backbone of my family and I didn’t dare to drag my body. I would not go to the pharmacy by myself unless the doctor prescribed medication to me. Anyway, there is medical insurance. It is better to go to a big hospital and feel at ease.”

Income level is an important factor. Compared to the lowest income group (<1000 Yuan), those with a monthly income of 1000 to 1900 Yuan have a higher possibility of going to a pharmacy rather than a hospital (OR = 6.92, 95%CI:1.20, 39.90). A respondent answered: “the fundamental influencing factor for ordinary people to see a doctor is money. If you have money, you can go to a good hospital, and if you don’t have money, you can go to a lower level hospital. No matter what age you are, money is the most important thing”. Another said: “for general diseases, wealthy elderly people will also go to good hospitals to see a doctor. They are not short of money, but for ordinary families, they may not go to good hospitals. But it is not the same for major diseases. If it is a serious disease that determines life and death. Money shouldn’t matter. It’s important to save your life!” The lowest-income people generally have a minimum level of income security and the government gives subsidies when they go to the hospital. Because of their understanding of health and sufficient financial resources, the highest-income group can afford the hospital whereas the middle-income group choose to go to the pharmacy given cost pressures. Compared with those who are married with a spouse, single people are more willing to go to the pharmacy (OR = 17.85, 95%CI: 1.37, 232.55). Compared with elderly couples living together, those who live alone are more willing to take other measures (OR = 97.47, 95%CI: 2.70, 3518.98).

Whether elderly people have Shanghai *hukou* also affects their choice in seeking medical help in the condition of physical discomfort. Compared with elderly without Shanghai *hukou*, those with Shanghai *hukou* may be less willing to go to the pharmacy (OR = 0.11, 95%CI: 0.02, 0.49) or will endure at home (OR = 0.06, 95%CI: 0.00, 0.68). Elderly people who do not have Shanghai *hukou* will have to pay the medical expenses in full once they seek medical treatment, whether they are outpatients or inpatients. Even if they have a permanent residence permit or medical insurance in other places, they still cannot enjoy the same benefits as those with Shanghai *hukou*. Therefore, it is clear that the elderly with Shanghai *hukou* enjoy an advantage in the use of public health resources.

Factors, such as education level and belonging to a rural or urban community, are not significant when the elderly are in physical discomfort.

Second, in terms of medical needs, elderly people with chronic disease have a higher possibility of going to a hospital instead of going to a pharmacy (OR = 0.54, 95%CI: 0.31, 0.93) or taking other measures (OR = 0.11, 95%CI: 0.02, 0.84). In recent years, in order to improvement chronic disease management, China has implemented a series of policies to control chronic disease prevalence and mortality, such as family doctor services, more convenient payment, and others. Hospitals are gradually making physical examinations more standardized, carrying out high-risk group screening and interventions and strengthening early detection and treatment of major chronic diseases [36].

Third, in terms of medical resource availability, when in physical discomfort, the subjective and objective distances of the elderly person’s residence to the nearest district hospital significantly influences their medical treatment behaviour. The subjective and objective distances of the elderly people’s residential location to the nearest general hospitals have no significant influence. Compared to the group living the shortest distance from a hospital (<1 km), those who live 1–2 km (OR = 0.01, 95%CI: 0.00, 0.48) and 2–5 km (OR = 0.002, 95%CI: 0.00, 0.57) away choose to go to the hospital instead of taking other measures. Those who live 5–10 km away choose to endure at home (OR = 44.07, 95%CI: 0.66, 2920.79) and those who are 10 km and above from a hospital choose to go to the hospital instead of the pharmacy (OR = 0.22, 95%CI: 0.04, 1.32). From the objective distance perspective, we verified that those who live 1–2 km (OR = 0.15, 95%CI: 0.03, 0.76) and 2–5 km (OR = 0.05, 95%CI: 0.00, 1.13) away choose to go to the hospital instead of enduring at home. Those who live 5 km and above away choose to go to the hospital instead of the pharmacy (OR = 0.16, 95%CI: 0.03, 0.91). Other groups who are located further away from the hospital are more likely to go to hospital when experiencing physical discomfort rather than going to the pharmacy or enduring at home. This means that compared with elderly patients and their family members who live far away, elderly people who live nearby can afford to take on more risks as they have ample time and opportunity.

Fourth, in terms of medical insurance reimbursement policy, compared with those who do not understand the policy, those who understand the policy have a higher possibility of going to the hospital, rather than enduring at home (OR = 0.26, 95%CI:0.07, 0.93). When experiencing physical discomfort, elderly people who are unsure whether they prefer to go to institutions with a higher medical insurance reimbursement rate have a higher possibility of taking other measures (OR = 7.86, 95%CI: 0.92, 67.02). This means that people who are not sensitive to cost exclude hospital visits. This is mainly due to asymmetric information. The elderly population have two kinds of medical preferences. The first is that they prefer to choose medical treatment according to past experiences. They believe that taking other measures is better for them to resolve their illness. They also prefer the same medical treatment choice that they previously made. The second one is that elderly people often live alone and lack care from their children. They are afraid that the complicated medical treatment process could be a burden to their children, cannot understand their medical insurance reimbursement policy, and have information bias. An interviewee said: “major illnesses are basically treated in county hospitals or hospitals in the city. It would be a hassle to go to these hospitals for follow-up treatment. It takes an hour to get to the county hospital by bus from the village, let alone the city. The follow-up treatment must be transferred to the town and we won’t stay in the big hospital. When we go by ourselves, the children are not comfortable so they accompany us to see a doctor. This prevents them from making money. Also, hospitalization and medicine is expensive in big hospitals.”

### 3.3. The Influencing Factors of Medical Institution Choices in the Condition of General Disease and Severe Disease

Table 3 contains the influencing factors for general and severe disease. For general disease, compared with elderly people aged 60–64, those aged 75–79 are more willing to choose a district hospital (OR = 2.83, 95%CI: 0.87, 9.24). Compared with married couples, a divorced elderly person is more willing to go to the general hospital (OR = 0.18, 95%CI: 0.03, 0.98). Single people are more concerned about whether they can get quality and standard care to recover. Compared with elderly couples living together, elderly people living with children (OR = 2.29, 95%CI:1.04, 5.07) or grandchildren (OR = 2.59, 95%CI:0.89, 7.55) are more willing to go to district hospitals. A reasonable explanation is that those who only live with children may be younger. Some factors, such as gender, income, education level, rural or urban, and *hukou*, are not significant.

A healthy elderly individual is more willing to go to a district hospital (OR = 2.12, 95%CI:1.10, 4.06) as well as specialised hospitals or community clinics (OR = 1.79, 95%CI: 0.93, 3.46). Some special types of medicines are only dispensed in general hospitals and some can meet their daily needs only with medicines available from a community clinic.

The influence of the person’s subjectively perceived distance from home to hospital is not significant; however, the objective distance is significant. Compared with those whose hospitals are objectively closest to their homes (district hospital), those who live near general hospitals have a higher possibility of going to tertiary hospitals rather than secondary hospitals (OR = 0.32, 95%CI: 0.10, 1.00) or specialised hospitals, or community clinics (OR = 0.19, 95%CI:0.05, 0.73). Compared with the elderly who are objectively closest to the general hospital (<1 km), those who are objectively 1–2 km away are more willing to go to the district or regional hospital (OR = 3.21, 95%CI: 0.88, 11.7) or specialised hospitals or community clinics (OR = 5.17, 95%CI: 1.20, 22.32). The elderly who are 2–5 km away are more willing to visit specialised hospitals or community clinics (OR = 44.85, 95%CI: 2.78, 724.03). This means that patients who are closer to a tertiary hospital are more willing to take risks. Compared with those who are objectively closest to the district hospitals (<1 km), those whose objective distance is 1–2 km from the district hospital tend to go to a district hospital rather than the general hospital (OR = 2.60, 95%CI: 0.97, 6.99). The elderly who live 2–5 km (OR = 3.35, 95%CI: 0.81, 13.88) and over 5 km (OR = 6.52, 95%CI: 0.82, 51.57) away are more willing to go to specialised hospitals or community clinics instead of a general hospital. The further they live from a general hospital, the higher the possibility that they will visit a district hospital or community clinic. The further they live from a district hospital, the higher the possibility that they will visit secondary hospitals. This is not a contradictory conclusion because those communities close to secondary hospitals are mostly in the suburbs so there are less options to choose from.

According to the current hierarchical diagnosis and treatment system and medical insurance reimbursement system in Shanghai, the higher level a hospital is, the lower medical insurance reimbursement rate it has. Compared with elderly people who tend to go to hospitals with high reimbursement rates, elderly people who do not go are more willing to choose general hospitals instead of specialised hospitals or community clinics (OR = 0.46, 95%CI: 0.22, 0.95).

For severe disease, compared with elderly people aged 60–64, elderly people aged 75–79 are more willing to choose a district hospital (OR = 2.95, 95%CI: 0.88, 9.92). This may be because the older a person is, the more they prefer conservative medical treatment so the possibility of going to a general hospital is lower. Compared with the lowest income group (<1000 yuan a month), those with a monthly income of 1000–1999 yuan are more willing to go to a general hospital (OR = 0.21, 95%CI: 0.03, 1.23). This also applies to those earning 2000–2999 yuan a month (OR = 0.13, 95%CI: 0.02, 1.06) and 5000–5999 yuan a month (OR = 0.07, 95%CI: 0.01, 0.71). Compared with an elderly couple living together, elderly people living with grandchildren are more willing to go a general hospital than a district or regional hospital (OR = 0.30, 95%CI: 0.08, 1.07). Their medical treatment behaviour is positively influenced by their children who provide financial and emotional support. Compared with those living in a rural community, those who live in an urban community are more willing to go to a general hospital than a district hospital (OR = 0.17, 95%CI: 0.02, 1.28). This is likely due to the fact that general hospital accessibility in urban areas is easier.

Compared with very healthy elderly people, unhealthy elderly people are more willing to go to a general hospital rather than a specialised hospital or community clinic (OR = 0.09, 95%CI: 0.01, 0.8). Chronic disease is not significant.

From a macro perspective, the distance of the residential location to a hospital significantly influences elderly people’s medical treatment behaviour. Compared with those subjectively closest to the general hospital (<1 km), those whose subjective distance from the general hospital is 1–2 km (OR = 0.05, 95%CI: 0, 0.67) and 2–5 km (OR = 0.07, 95%CI:0.01, 0.73) tend to go to the general hospital instead of a district hospital. Those whose subjective distance is more than 10 km from the closest tertiary hospitals are more willing to go to a general hospital instead of specialised hospitals or community clinics (OR = 0.07, 95%CI: 0, 1.54).

Compared with those who are subjectively closest to the district hospital (<1 km), those whose subjective distance is 1–2 km (OR = 0.24, 95%CI: 0.06, 0.96), 2–5 km (OR = 0.12, 95%CI: 0.02, 0.61), and over 10 km (OR = 0.17, 95%CI: 0.02, 1.24) away tend to go to the general hospital instead of a district hospital.

Compared to those who are objectively closest to the district hospitals (<1 km), those who are objectively 2–5 km away are more willing to visit a specialised hospital or community clinic (OR = 4.59, 95%CI: 1, 20.96) instead of going to a general hospital. This demonstrates that for severe disease, generally, regardless of the distance, the preference is to visit a general hospital.

## 4. Discussion

Seeing a doctor is a decision made by a person on the basis of physical condition, affordability, access to medical resources, understanding of the disease, psychological expectations, family support, and social environment. This is a complex and systematic process. This paper verifies that the causes of differences in the elderly population’s medical treatment behaviour are complex and are affected by individual characteristics, socioeconomic characteristics, medical needs, medical resource availability, and medical expenditure etc.

However, some similarities and differences between this and other studies should be emphasised. First, scholars have separately verified the important influence of education level, marital status, living habits, socioeconomic status, etc. Education plays a role in elderly medical behaviour [12], but this article does not support this. At the same time, this article also finds that the influence of marriage on elderly behaviour is based on the severity of the illness and seeking medical treatment is not only an individual behaviour, but also that of a family and society. It is a kind of rational choice. For example, depending on the severity of the illness, the impact of socioeconomic status is different. As the severity of the disease increases, the willingness to pay for medical treatment of the high-income elderly group increases faster than the low-income elderly group. This confirms that the behaviour of the elderly also follows the view of the classic medical behaviour model: people with different socioeconomic levels and demographic characteristics have systematic differences in the utilisation of medical and health services [37]. However, when the elderly face physical discomfort or general disease, the role of socioeconomic factors is less obvious. Part of the reason is that Shanghai’s elderly people have high income levels and social security and there is no significant difference in medical expenditure for general diseases. From the micro perspective of families in cities, families will provide great support for critically ill patients. This improves the health of the elderly and intergenerational family relationships (patients can provide retirement support to the family after they recover).

Second, the impact of China’s medical marketisation reform on health equity is not very favourable. On the one hand, the reform of public hospitals in China requires not only hospitals to play a role in public welfare, but also allows doctors to increase hospital revenue through drug bonuses and equipment use. As a result, tertiary hospitals are overcrowded and market-oriented health reform has not only failed to solve the problem of rising medical expenses, but also reduced the access to health services of the most vulnerable people. On the other hand, middle-income elderly groups are not included in a medical assistance plan and in the event of market failure, medical insurance cannot relieve the payment pressure faced by most middle-income elderly groups. In addition, the reimbursement for medical treatment in Shanghai requires the insured person to register and file for medical treatment in a cross-provincial region in accordance with the relevant regulations of the insured’s place and to choose a hospital that has opened a national direct settlement system for medical treatment. Some elderly people do not understand the reimbursement policy when seeking medical treatment and are still affected by the divisions of the medical insurance system.

Third, when the illness is severe, the spatial allocation of medical resources has a significant impact on the preference of the elderly population in China’s large cities. There is also a distance friction effect in medical treatment: the closer the objective distance to the hospital, the stronger the tendency to choose. The influence of a general hospital is especially obvious. However, for general disease, the distance from the district hospital (secondary hospital) or the general hospital (tertiary hospital) does not affect their choice to visit the district hospital. Because Shanghai has a relatively high rate of health awareness and knowledge amongst the elderly, the queue for secondary hospitals is generally better than that of a general hospital so there is no need to choose crowded tertiary hospitals for general disease. In the case of the severe disease, the elderly are reluctant to go to a district hospital because they know that the classification of Chinese hospitals relates to the quality of doctors and the hospital.

Our results call for stronger government support of elderly medical resource system design. First, medical supply should be reformed according to elderly population demand characteristics to avoid any ‘one-size-fits-all’ strategies. It should be provided with targeted medical services based on people’s characteristics and needs. Second, it would be appropriate to establish medical insurance that suits the needs of the market. However, there are still flaws in the design and implementation of the medical insurance system. A reasonable reimbursement ratio should be designed according to the severity of the illness and the type of the disease rather than household registration status or hospital level. Third, the government urgently needs to pay attention to the “centripetal” gathering of public resources, such as medical care, and the “centrifugal” spread of the elderly population. In the suburbs of Shanghai, especially in the outer suburbs, there is a lack of higher quality medical resources and even basic hospitals and community clinics are scarce. This has caused the allocation of medical and health resources to be deemed as “average” but not “equal”.

This study has some limitations. First, it only considered the retirement pension received rather than accumulated wealth. Second, studies have shown that the financial support received by one’s children directly improves the utilisation and affordability of medical services. This indicates other potential available medical service resources for the elderly [38], but the sample size of the elderly who receive financial support from their children in this study is limited so it may not have been adequately studied.

With the deepening of China’s medical marketization reform, the universal free medical system in the era of planned economy that emphasises fair and low-level universal protection is moving towards the market economy era of the medical insurance system, which emphasises efficiency first but takes into account fair multi-level and multi-system medical insurance protection. In the process of continuing to learn from the Western medical system, the differences between the Chinese and Western medical security systems are constantly shrinking, and both are constantly reforming in the direction of marketization while taking into account fairness. In future research, based on the comparison of the differences between Chinese and Western medical security systems, the theory and analysis framework of the influence mechanism of medical behaviour choices for the elderly in the “Chinese context” should be proposed.

## 5. Conclusions

The results of the study conducted show the following. First, the medical behaviour of the elderly is a rational decision made under the influence of personal, family, and social conditions. The level of influence of each factor differs depending on the medical condition experienced. In this new stage of medical reform, we should pay more attention to the elderly, not only towards strengthening the support provided for this vulnerable group, but also to the adverse consequences that income stratification, health stratification, and general group differences have on the elderly population. In particular, we should pay special attention to the medical support provided to the middle- and low-income population suffering from major diseases. Second, China’s medical insurance system needs to be designed more thoroughly and should focus on the core incentive policy of “improving the quality of medical services and paying attention to the needs of patients”. The health insurance system could benefit from setting reimbursement rates based on a patient’s condition and disease type. Third, medical behaviour has a distance friction effect, but the allocation of public service resources still shows a strong centripetal concentration. The rural elderly population’s access to comprehensive medical treatment within the suburbs of big cities is low and it is necessary to take measures regarding the regional distribution of the elderly in big cities and promote the fair distribution of medical resources in Shanghai. Our findings contribute to the understanding of medical needs of various types of elderly groups. It also sheds light on how to help reduce medical problems encountered by the elderly population. More generally, it also provides theoretical support and empirical evidence for urban public health policies in developing countries.

## Figures and Tables

**Table 1:** The elderly population’s choices made in the condition of physical discomfort (%).

Characteristics	Going to Hospital N = 284 (64.69%)	Going to Pharmacy N = 111 (25.28%)	Enduring N = 30 (6.83%)	Other Measures N = 14 (3.19%)
**Gender**				
Male	36.16	38.16	25.53	36.00
Female	63.84	61.84	74.47	64.00
**Age**				
60–64 years old	25.69	36.84	27.66	40.00
65–69 years old	31.17	30.92	29.79	36.00
70–74 years old	20.70	15.13	25.53	-
75–79 years old	11.22	8.55	12.77	16.00
80 years old and above	11.22	8.56	4.25	8.00
**Income (retirement pension)**				
<1000 Yuan	4.75	4.61	6.38	-
1000–1999 Yuan	15.50	24.34	10.64	8.00
2000–2999 Yuan	7.25	15.79	10.64	4.00
3000–3999 Yuan	40.75	30.26	57.45	32.00
4000–4999 Yuan	18.50	14.47	8.50	28.00
5000–5999 Yuan	6.25	5.92	2.13	12.00
6000 Yuan and above	7.00	4.61	4.26	16.00
**Marital status**				
Unmarried	0.25	3.31	2.13	16.00
Married	81.05	82.12	-	-
Divorced	2.49	1.32	80.85	68.00
Widowed	16.21	13.25	17.02	16.00
**Cohabitation status**				
Living alone	8.24	7.89	10.64	24.00
Living with spouse	51.87	46.05	42.55	36.00
With elder (same generation)	2.74	2.64	8.51	8.00
With children	26.68	26.97	29.79	28.00
With three generations	10.47	16.45	8.51	4.00
**Education level**				
Primary school and below	19.00	27.63	19.15	16.00
Middle school	43.75	42.76	40.43	24.00
High school or technical secondary school	25.50	19.08	21.28	36.00
Junior college	11.75	10.53	19.14	24.00
**Rural or Urban**				
Urban	89.45	84.21	95.74	92.00
Rural	10.55	15.79	4.26	8.00
**Shanghai *Hukou***				
Yes	97.49	90.73	93.62	88.00
No	2.51	9.27	6.38	12.00
**Health condition**				
Healthy	42.00	53.29	29.79	48.00
Basic level of health	45.00	33.55	51.06	48.00
Unhealthy	13.00	13.16	19.15	4.00
**Chronic disease**				
No	28.00	38.82	27.66	56.00
Yes	72.00	61.18	72.34	44.00
**The level of the subjectively nearest hospital from home**				
level-2	42.78	48.23	41.03	42.11
level-3	57.22	51.77	58.97	57.89
**The subjective distance to the nearest hospital**				
<1 km	5.71	6.90	8.83	20.00
1–2 km	27.31	28.45	11.76	20.00
2–5 km	37.78	31.90	58.83	33.33
5–10 km	8.57	6.03	8.83	6.67
10 km and above	20.63	26.72	11.76	20.00
**The level of the objectively nearest hospital from home**				
level-2	75.31	73.03	76.60	76.00
level-3	24.69	26.97	23.40	24.00
**The objective distance to the nearest level-3 hospital**				
<1 km	37.40	40.13	55.32	28.00
1–2 km	33.67	29.61	25.53	40.00
2–5 km	16.96	22.37	17.02	24.00
5 km and above	11.97	7.89	2.13	8.00
**Understand medical insurance reimbursement policy**				
Yes	66.17	65.79	87.23	88.00
No	33.83	34.21	12.77	12.00
**Prefer medical institutions with higher reimbursement rate**				
Yes	39.25	42.76	29.79	28.00
No	32.75	30.92	31.91	32.00
Not sure	28.00	26.32	38.30	40.00

Note: - Represents no observations here.

**Table 2:** Parameter estimation for the elderly population in the condition of physical discomfort (control group = going to hospital).

Characteristics	Going to Pharmacy		Enduring		Other Measures	
	Odds Ratio	95%CI	Odds Ratio	95%CI	Odds Ratio	95%CI
**Demographic and economic characteristics** **:**						
**Gender**						
Male	Reference		Reference		Reference	
Female	0.76	(0.40, 1.45)	0.65	(0.21, 2.01)	0.90	(0.07, 11.05)
**Age**						
60–64 years old	Reference		Reference		Reference	
65–69 years old	0.72	(0.37, 1.38)	0.64	(0.18, 2.18)	0.25	(0.02, 2.9)
70–74 years old	0.46 *	(0.20, 1.06)	0.61	(0.15, 2.46)	0.74	(0.05, 11.87)
75–79 years old	0.59	(0.22, 1.55)	1.32	(0.27, 6.53)	0.00	(0.00, .)
80 years old and above	0.27 **	(0.09, 0.88)	0.17	(0.01, 2.20)	0.19	(0.00, 10.77)
**Income** **(retirement pension)**						
<1000 Yuan	Reference		Reference		Reference	
1000–1999 Yuan	6.92 **	(1.20, 39.97)	1.68	(0.07, 40.69)	7.25 × 10^6^	(0.00, .)
2000–2999 Yuan	2.02	(0.35, 11.73)	2.45	(0.11, 53.73)	2.70 × 10^7^	(0.00, .)
3000–3999 Yuan	1.07	(0.20, 5.79)	1.80	(0.09, 36.34)	7.41 × 10^8^	(0.00, .)
4000–4999 Yuan	1.06	(0.18, 6.31)	0.62	(0.02, 16.46)	7.32 × 10^8^	(0.00, .)
5000–5999 Yuan	1.10	(0.17, 7.29)	0.52	(0.01, 19.53)	7.23 × 10^9^	(0.00, .)
6000 Yuan and above	0.78	(0.11, 5.76)	0.68	(0.02, 21.91)	2.09 × 10^9^	(0.00, .)
**Marital status**						
Unmarried	17.85 **	(1.37, 232.55)	0.00	(0, .)	13.71	(0.11, 1739.55)
Married	Reference		Reference		Reference	
Divorced	0.15	(0.02, 1.48)	0.00	(0.00, .)	0.00	(0.00, .)
Widowed	0.99	(0.35, 2.77)	0.61	(0.09, 4.27)	0.15	(0.00, 7.09)
**Cohabitation status**						
Living alone	1.31	(0.36, 4.83)	4.70	(0.50, 44.18)	97.47 **	(2.70, 3518.98)
Living with spouse	Reference		Reference		Reference	
With elder (same generation)	0.96	(0.18, 5.16)	2.01	(0.22, 18.67)	4.99	(0.1, 253.48)
With children	1.25	(0.65, 2.41)	0.73	(0.20, 2.67)	3.83	(0.5, 29.16)
With three generations	1.18	(0.51, 2.71)	0.71	(0.16, 3.23)	0.00	(0.00, .)
**Education level**						
Primary school and below	Reference		Reference		Reference	
Middle school	0.72	(0.33, 1.57)	0.44	(0.10, 1.92)	0.37	(0.01, 9.86)
High school or technical secondary school	0.47	(0.19, 1.20)	0.69	(0.14, 3.41)	0.37	(0.01, 10.31)
Junior college	0.83	(0.27, 2.59)	1.27	(0.18, 8.99)	1.61	(0.03, 75.17)
**Rural or Urban**						
Urban	1.02	(0.18, 5.61)	1.01	(0.03, 36.54)	0.04	(0.00, 390.24)
Rural	Reference		Reference		Reference	
**Shanghai *Hukou***						
Yes	0.11 ***	(0.02, 0.49)	0.06 **	(0.00, 0.68)	4080.37	(0.00, .)
No	Reference		Reference		Reference	
**Medical demand characteristics** **:**						
**Health condition**						
Healthy	Reference		Reference		Reference	
Basic level of health	1.04	(0.60, 1.81)	1.07	(0.37, 3.07)	1.95	(0.30, 12.51)
Unhealthy	1.15	(0.51, 2.59)	2.23	(0.63, 7.94)	5.35	(0.18, 156.41)
**Chronic disease**						
No	Reference		Reference		Reference	
Yes	0.54 **	(0.31, 0.93)	0.74	(0.26, 2.09)	0.11 **	(0.02, 0.84)
**Medical resource availability** **:**						
**The level of the subjectively nearest hospital from home**						
level-2	Reference		Reference		Reference	
level-3	0.57	(0.06, 5.23)	0.00	(0.00, .)	0.04	(0.00,21.82)
**The subjective distance to the nearest hospital**						
Distance from Level-3 hospital <1 km	Reference		Reference		Reference	
1–2 km	0.66	(0.10, 4.53)	1.36 × 10^7^	(0.00, .)	0.04	(0.00, 52.06)
2–5 km	0.91	(0.14, 5.76)	5.24 × 10^7^	(0.00, .)	1.04	(0.00, 326.2)
5–10 km	0.31	(0.04, 2.59)	1.21 × 10^7^	(0.00, .)	0.00	(0.00, .)
10 km and above	0.51	(0.06, 3.99)	2.45 × 10^7^	(0.00, .)	3.66	(0.01, 1371.82)
Distance from Level-2 hospital <1 km	Reference		Reference		Reference	
1–2 km	0.93	(0.24, 3.55)	0.00	(0.00, .)	0.01 **	(0.00, 0.48)
2–5 km	0.31	(0.07, 1.48)	1.62	(0.18, 14.39)	0.00 **	(0.00, 0.57)
5–10 km	0.27	(0.02, 3.94)	44.07*	(0.66, 2920.79)	37.07	(0.09, 14,913.39)
10 km and above	0.22 *	(0.04, 1.32)	2.11	(0.08, 55.02)	0.06	(0.00, 6.64)
**The level of the objectively nearest hospital from home**						
level-2	Reference		Reference		Reference	
level-3	1.36	(0.44, 4.15)	1.09	(0.20, 6.10)	0.00	(0.00, .)
**The objective distance to the nearest level-3 hospital**						
Distance from Level-3 hospital <1 km	Reference		Reference		Reference	
1–2 km	0.75	(0.21, 2.65)	0.31	(0.03, 2.82)	2.17 × 10^7^	(0.00, .)
2–5 km	0.56	(0.07, 4.56)	0.16	(0.00, 5.72)	2.91 × 10^7^	(0.00, .)
5 km and above	0.00	(0.00, .)	0.00	(0.00, .)	1.84 × 10^6^	(0.00, .)
Distance from Level-2 hospital <1 km	Reference		Reference		Reference	
1–2 km	0.85	(0.36, 2.00)	0.15 **	(0.03, 0.76)	1.11	(0.07, 18.32)
2–5 km	1.97	(0.54, 7.18)	0.05 *	(0.00, 1.13)	1.28	(0.04, 37.65)
5 km and above	0.16**	(0.03, 0.91)	0.09	(0.00, 2.70)	0.00	(0.00, .)
**Medical expenditure** **:**						
**Understand medical insurance reimbursement policy**						
Yes	0.87	(0.50, 1.52)	0.26 **	(0.07, 0.93)	0.16	(0.01, 2.00)
No	Reference		Reference		Reference	
**Prefer medical institutions with higher reimbursement rate**						
Yes	Reference		Reference		Reference	
No	0.96	(0.52, 1.77)	1.48	(0.44, 4.95)	0.70	(0.05, 10.24)
Not sure	0.86	(0.46, 1.62)	2.37	(0.77, 7.31)	7.86 *	(0.92, 67.02)
**Constant**	20.51 **	(1.02, 411.47)	11.11	(0.06, 2029.61)	0.00	(0.00, .)
**Observations**	439		439		439	
**r2_p**	0.219		0.219		0.219	

Note: *, **, and *** respectively represent significance at 10%, 5%, and 1%.

**Table 3:** Parameter estimation for medical institution choices of an elderly population with general disease (control group = general hospitals).

	General Disease				Severe Disease			
Characteristics	District Hospital		Other Specialised Hospitals or Community Clinics		District Hospital		Other Specialised Hospitals or Community Clinics	
	Odds Ratio	95%CI	Odds Ratio	95%CI	Odds Ratio	95%CI	Odds Ratio	95%CI
**Demographic and economic characteristics:**								
**Gender**								
Male	Reference		Reference		Reference		Reference	
Female	1.73	(0.82, 3.67)	1.37	(0.64, 2.90)	1.59	(0.72, 3.49)	0.97	(0.42, 2.21)
**Age**								
60–64 years old	Reference		Reference		Reference		Reference	
65–69 years old	0.65	(0.30, 1.40)	0.54	(0.25, 1.17)	0.73	(0.31, 1.71)	0.55	(0.23, 1.31)
70–74 years old	1.68	(0.65, 4.31)	1.98	(0.77, 5.12)	1.31	(0.49, 3.5)	0.92	(0.34, 2.48)
75–79 years old	2.83 *	(0.87, 9.24)	1.43	(0.41, 4.97)	2.95*	(0.88, 9.92)	1.48	(0.47, 4.65)
80 years old and above	0.77	(0.21, 2.85)	1.42	(0.41, 4.84)	2.14	(0.58, 7.95)	0.26	(0.04, 1.75)
**Income (retirement pension)**								
<1000 Yuan	Reference		Reference		Reference		Reference	
1000–1999 Yuan	0.54	(0.04, 8.09)	0.47	(0.03, 7.24)	0.21 *	(0.03, 1.23)	1.27 × 10^6^	(0.00, .)
2000–2999 Yuan	0.27	(0.02, 3.46)	0.19	(0.01, 2.59)	0.13 *	(0.02, 1.06)	2.53 × 10^6^	(0.00, .)
3000–3999 Yuan	0.71	(0.06, 8.79)	0.76	(0.06, 9.82)	0.43	(0.07, 2.52)	1.88 × 10^6^	(0.00, .)
4000–4999 Yuan	0.47	(0.04, 6.01)	0.40	(0.03, 5.39)	0.26	(0.04, 1.72)	1.02 × 10^6^	(0.00, .)
5000–5999 Yuan	0.32	(0.02, 4.45)	0.27	(0.02, 3.97)	0.07 **	(0.01, 0.71)	6.80 × 10^6^	(0.00, .)
6000 Yuan and above	0.39	(0.03, 5.81)	0.31	(0.02, 4.89)	0.36	(0.04, 3.11)	1.19 × 10^5^	(0.00, .)
**Marital status**								
Unmarried	0.67	(0.06, 7.20)	1.14	(0.09, 15.29)	0.64	(0.04, 10.97)	1.08	(0.13, 8.90)
Married	Reference		Reference		Reference		Reference	
Divorced	0.18**	(0.03, 0.98)	0.11 **	(0.01, 0.78)	3.23	(0.58, 17.89)	0.45	(0.04, 4.61)
Widowed	0.42	(0.13, 1.33)	0.60	(0.19, 1.94)	0.55	(0.14, 2.22)	0.88	(0.26, 3.02)
**Cohabitation status**								
Living alone	2.18	(0.52, 9.19)	2.26	(0.55, 9.19)	0.91	(0.17, 4.8)	1.43	(0.32, 6.32)
Living with spouse	Reference		Reference		Reference		Reference	
With elder (same generation)	1.22	(0.25, 6.02)	0.30	(0.05, 1.92)	1.27	(0.12, 13.07)	2.02	(0.43, 9.61)
With children	2.29**	(1.04, 5.07)	1.28	(0.57, 2.85)	1.45	(0.6, 3.47)	1.30	(0.58, 2.93)
With three generations	2.59*	(0.89, 7.55)	2.15	(0.72, 6.44)	0.30 *	(0.08, 1.07)	1.20	(0.36, 4.08)
**Education level**								
Primary school and below	Reference		Reference		Reference		Reference	
Middle school	0.87	(0.32, 2.35)	1.55	(0.56, 4.24)	0.77	(0.30, 1.95)	0.99	(0.32, 3.03)
High school or technical secondary school	0.70	(0.24, 2.05)	0.87	(0.29, 2.62)	0.90	(0.30, 2.71)	0.80	(0.22, 2.90)
Junior college	1.07	(0.30, 3.85)	1.29	(0.34, 4.81)	1.13	(0.30, 4.27)	2.18	(0.54, 8.72)
**Rural or Urban**								
Urban	0.33	(0.03, 3.21)	0.59	(0.06, 5.78)	0.17 *	(0.02, 1.28)	6.18	(0.47, 81.41)
Rural	Reference		Reference		Reference		Reference	
***Hukou***								
Yes	0.58	(0.07, 5.02)	0.52	(0.06, 4.75)	0.46	(0.08, 2.64)	2.65 × 10^6^	(0.00, .)
No	Reference		Reference		Reference		Reference	
**Medical demand characteristics:**								
**Health condition**								
Healthy	Reference		Reference		Reference		Reference	
Basic level of health	2.12 **	(1.10, 4.06)	1.79 *	(0.93, 3.46)	1.24	(0.62, 2.48)	0.97	(0.50, 1.88)
Unhealthy	1.30	(0.48, 3.49)	1.62	(0.61, 4.30)	0.99	(0.35, 2.75)	0.09 **	(0.01, 0.80)
**Chronic disease**								
No	Reference		Reference		Reference		Reference	
Yes	0.95	(0.49, 1.84)	0.72	(0.37, 1.39)	1.31	(0.63, 2.73)	0.67	(0.33, 1.34)
**Medical resource availability:**								
**The level of the subjectively nearest hospital from home**								
level-2	Reference		Reference		Reference		Reference	
level-3	0.00	(0.00, .)	0.00	(0.00, .)	2.29	(0.18, 29.03)	4.23	(0.15, 123.22)
**The subjective distance to the nearest hospital**								
Distance from Level-3 hospital <1 km	Reference		Reference		Reference		Reference	
1–2 km	1.42	(0.16, 12.62)	0.44	(0.05, 3.72)	0.05 **	(0.00, 0.67)	0.32	(0.02, 4.58)
2–5 km	1.46	(0.17, 12.27)	1.01	(0.13, 7.71)	0.07 **	(0.01, 0.73)	0.37	(0.03, 4.88)
5–10 km	0.92	(0.09, 9.55)	0.35	(0.04, 3.47)	0.13	(0.01, 1.66)	0.24	(0.01, 4.46)
10 km and above	1.27	(0.12, 13.23)	0.70	(0.07, 6.62)	0.16	(0.01, 1.94)	0.07 *	(0.00, 1.54)
Distance from Level-2 hospital <1 km	Reference		Reference		Reference		Reference	
1–2 km	0.00	(0.00, .)	0.00	(0.00, .)	0.24 **	(0.06, 0.96)	3.23	(0.34, 31.2)
2–5 km	0.00	(0.00, .)	0.00	(0.00, .)	0.12 **	(0.02, 0.61)	1.51	(0.13, 16.96)
5–10 km	0.00	(0.00, .)	0.00	(0.00, .)	5.05	(0.30, 84.72)	0.00	(0.00, .)
10 km and above	0.00	(0.00, .)	0.00	(0.00, .)	0.17 *	(0.02, 1.24)	1.62	(0.11, 23.88)
**The level of the objectively nearest hospital from home**								
level-2	Reference		Reference		Reference		Reference	
level-3	0.32 *	(0.1, 1)	0.19 **	(0.05, 0.73)	0.00	(0.00, .)	1.21	(0.31, 4.72)
**The objective distance to the nearest level hospital**								
Distance from Level-3 hospital <1 km	Reference		Reference		Reference		Reference	
1–2 km	3.21 *	(0.88, 11.70)	5.17 **	(1.2, 22.32)	4.65 × 10^6^	(0.00, .)	0.63	(0.13, 2.99)
2–5 km	6.10	(0.40, 94.03)	44.85 ***	(2.78, 724.03)	4.42 × 10^6^	(0.00, .)	0.00	(0.00, .)
5 km and above	10.78	(0.00, .)	0.00	(0.00, .)	1.58 × 10^16^	(0.00, .)	2.58 × 10^6^	(0.00, .)
Distance from Level-2 hospital <1 km	Reference		Reference		Reference		Reference	
1–2 km	2.60 *	(0.97, 6.99)	2.30	(0.84, 6.32)	0.81	(0.28, 2.37)	1.59	(0.58, 4.30)
2–5 km	1.18	(0.25, 5.49)	3.35 *	(0.81, 13.88)	0.42	(0.06, 3.18)	4.59 **	(1.00, 20.96)
5 km and above	4.49	(0.57, 35.40)	6.52 *	(0.82, 51.57)	0.76	(0.09, 6.43)	3.36	(0.40, 28.24)
**Medical expenditure:**								
**Understand medical insurance reimbursement policy**								
Yes	1.10	(0.56, 2.16)	1.20	(0.61, 2.34)	1.68	(0.85, 3.29)	1.78	(0.83, 3.80)
No	Reference		Reference		Reference		Reference	
**Prefer medical institutions with higher reimbursement rate**								
Yes	Reference		Reference		Reference		Reference	
No	0.76	(0.37, 1.57)	0.46 **	(0.22, 0.95)	1.30	(0.63, 2.69)	0.85	(0.38, 1.93)
Not sure	0.76	(0.35, 1.61)	0.62	(0.29, 1.31)	0.53	(0.23, 1.23)	1.37	(0.63, 2.98)
**Constant**	3.82 × 10^7^	(0.00, .)	2.59 × 10^7^	(0.00, .)	18.82 *	(0.61, 581.28)	0.00	(0.00, .)
**Observations**	439		439		439		439	
**r2_p**	0.147		0.147		0.21		0.21	

Note: *, **, and *** respectively represent significance at 10%, 5%, and 1%.

## Data Availability

The data presented in this study are available on request from the corresponding author. Second-hand data used comprised mainly of statistical data, government development reports etc., derived from China National Bureau of Statistics (http://www.stats.gov.cn/), Shanghai Municipal Peoples Government (http://www.shanghai.gov.cn/nw39426/20200821/0001-39426_50911.html, 1 January 2017) and Shanghai Bureau of Statistics (http://tjj.sh.gov.cn/),The contents of medical insurance policy in the questionnaire come from Shanghai Medical Security Bureau (http://ybj.sh.gov.cn/gfxwj3/index.html, 14 October 2019). Objective distance of residents from the hospital comes from (https://map.baidu.com/).

## Supplementary Materials

The following are available online at https://www.mdpi.com/article/10.3390/ijerph18084108/s1, File S1: Questionnaire: Shanghai elderly medical demand characteristics survey form.

## Abbreviations

CHN	China
MLR	Multinomial Logistic Regression
CHNS	China Health and Nutrition Survey
NHS	National Health Service
IIA	Independence of Irrelevant Alternatives
MLE	Maximum Likelihood Estimation

## Appendix A

**Table A1:** The elderly population’s options taken with general disease and severe disease (%).

Characteristics	General Disease			Severe Disease		
	General Hospital N = 97 (22.10%)	District Hospital N = 163 (37.13%)	Specialised Hospitals or Community Clinic N = 179 (40.78%)	General Hospital N = 312 (71.07%)	District Hospital N = 65 (14.81%)	Specialised Hospitals or Community Clinic N = 62 (14.12%)
**Gender**						
Male	46.83	34.22	32.23	35.71	40.40	31.52
Female	53.17	65.78	67.77	64.29	59.60	68.48
**Age**						
60–64 years old	30.16	29.33	28.21	28.34	28.28	33.70
65–69 years old	36.50	30.67	29.31	33.64	30.31	20.65
70–74 years old	14.29	19.11	22.34	18.66	21.21	21.74
75–79 years old	5.50	13.78	9.52	8.53	11.11	17.39
80 years old and above	13.49	7.11	10.62	10.83	9.09	6.52
**Income (retirement pension)**						
<1000 Yuan	3.17	3.11	6.62	4.61	6.12	3.26
1000–1999 Yuan	8.73	18.22	19.85	15.21	29.60	11.96
2000–2999 Yuan	11.11	9.33	8.82	8.53	11.22	11.96
3000–3999 Yuan	34.13	40.88	39.71	40.55	26.53	45.65
4000–4999 Yuan	23.02	17.33	14.34	17.05	16.33	18.48
5000–5999 Yuan	8.73	6.22	4.78	6.45	4.08	6.52
6000 Yuan and above	11.11	4.89	5.88	7.60	6.12	2.17
**Marital status**						
Unmarried	2.38	1.34	1.83	1.84	1.01	2.17
Married	75.40	81.69	82.42	79.91	85.86	79.35
Divorced	3.17	2.68	0.73	1.62	4.04	1.09
Widowed	19.05	14.29	15.02	16.63	9.09	17.39
**Cohabitation status**						
Living alone	7.94	8.44	9.89	9.45	5.05	10.87
Living with spouse	47.62	46.22	52.38	47.93	56.57	46.75
With elder (same generation)	5.55	3.11	2.56	2.99	3.03	5.43
With children	30.95	28.45	23.81	26.27	27.27	30.43
With three generations	7.94	13.78	11.36	13.36	8.08	6.52
**Education level**						
Primary school and below	14.40	23.56	21.98	19.17	32.32	17.39
Middle school	40.80	40.00	45.05	43.42	36.36	44.56
High school or technical secondary school	31.20	24.00	20.88	24.48	23.24	22.83
Junior college	13.60	12.44	12.09	12.93	8.08	15.22
**Rural or Urban**						
Urban	96.83	89.29	84.50	89.84	79.59	93.41
Rural	3.17	10.71	15.50	10.16	20.41	6.59
**Shanghai *Hukou***						
Yes	96.00	95.11	94.83	94.47	94.90	98.89
No	4.00	4.89	5.17	5.53	5.10	1.11
**Health condition**						
Healthy	53.97	40.63	42.49	42.49	45.46	50.00
Basic level of health	34.92	46.88	43.22	42.73	42.42	43.48
Unhealthy	11.11	12.49	14.29	14.78	12.12	6.52
**Chronic disease**						
no	33.60	31.56	31.14	30.48	30.30	39.13
Yes	66.40	68.44	68.86	69.52	69.70	60.87
**The level of the subjectively nearest hospital from home**						
level-2	31.58	48.28	46.47	40.75	50.60	53.95
level-3	68.42	51.72	53.53	59.25	49.40	46.05
**The subjective distance to the nearest hospital**						
<1 km	2.83	9.04	6.63	5.64	13.33	4.41
1–2 km	33.95	29.38	19.39	25.52	22.67	33.82
2–5 km	39.62	35.59	38.27	39.76	26.67	39.71
5–10 km	8.49	7.91	7.65	7.42	12.00	5.88
10 km and above	15.09	18.08	28.06	21.66	25.33	16.18
**The level of the objectively nearest hospital from home**						
level-2	65.08	77.78	76.92	70.97	80.81	86.96
level-3	34.92	22.22	23.08	29.03	19.19	13.04
**The objective distance to the nearest hospital**						
<1 km	51.59	39.56	32.97	39.41	35.35	41.30
1–2 km	30.16	36.00	30.03	35.48	24.24	26.09
2–5 km	11.11	14.22	25.64	17.74	21.22	19.57
5 km and above	7.14	10.22	11.36	7.37	19.19	13.04
**Understand medical insurance reimbursement policy**						
Yes	66.67	70.40	67.77	70.44	58.59	70.33
No	33.33	29.60	32.23	29.56	41.41	29.67
**Prefer medical institutions with higher reimbursement rate**						
Yes	33.33	35.71	43.95	37.79	45.45	37.36
No	38.10	33.93	28.21	32.72	33.33	28.57
Not sure	28.57	30.36	27.84	29.49	21.22	34.07
