# Supplementary material for: Medical Treatment Behaviour of the Elderly Population in Shanghai: Group Features and Influencing Factor Analysis
Source: Int J Environ Res Public Health. 2021 Apr 13;18(8):4108. doi: 10.3390/ijerph18084108 (PMC8070517; doi:10.3390/ijerph18084108)
Supplement: Supplementary file 1 [file ijerph-18-04108-s001.zip › Questionnaire.docx]

**Dear senior:**

Since our country's reform and opening up, with the transformation of the enterprise system and social security system, great changes have taken place in the medical system. Despite the great improvement in medical standards, residents still have great opinions on the medical service industry. At present, the government is trying to find ways to improve the medical supply of residents to meet the medical needs of the general population, especially the elderly.

Given that China has entered the elderly society, but the elderly are concerned about some issues that the society cannot solve well, such as medical problems. To this end, the Economic Development Institute of East China University of Science and Technology has specially established a research group on the medical needs of the elderly, hoping to provide practical and useful suggestions for the government to solve these problems through field investigations and research.

The information in this survey is for academic research reference only and has no commercial purpose. We guarantee that the specific situation of you and your family involved in this investigation will not be disclosed to the government, enterprises and any other third parties.

We sincerely hope for your strong support, and express our heartfelt thanks to you and your family for your support, and wish you good health and family happiness!

**Instructions for completing the form**

1. The questionnaire is filled in anonymously. There is no right or wrong answer to the question, please fill it out according to your situation.

2. Please carefully read the title and the option requirements after the question.

**Investigation notice**

1. The classification criteria for the elderly: regardless of the gender, the full age is over 60 years old (born before October 1, 1957);

2. If there are multiple elderly persons in a household, only one person will be investigated. Under the circumstances of choice, the survey subjects should choose people with high education level, or clear-minded and talkative people;

3. During the investigation, the elderly must be treated with great respect. There must be no abrupt behavior and disrespectful language. If the elderly feel physical discomfort or abnormal mood during the investigation, the investigation shall be terminated;

4. The information related to the questionnaire question obtained during the conversation, other circumstances of the investigator, and all information related to the subject of the survey should be recorded on the questionnaire paper as much as possible;

5. When the respondent encounters a problem that they do not understand, it is necessary to explain it as clearly as possible before allowing it to answer.

**Institute of Economic Development,**

**East China University of Science and Technology**

**November 01, 2017**

**Questionnaires coded:**

**Shanghai elderly medical demand characteristics survey form**

**Survey location： _________area________street________ community**

**Survey time：________**

**investigator：Name__________**

**1.Basic situation**

1. Your gender is？（ ）

A Male B Female

1. What is your age？（ ）

A 60-69 years old B 70-79 years old C 80 years old and above

1. Your education level？（ ）

A Have not been to school B Primary school C Junior high school D High school or Technical secondary school E College and above

1. What is your current marital status? （ ）

A Unmarried B Married with spouse C Divorced D Widowed

1. Your relationship with co-residents (multiple choices) ( )

A Spouse B Children C Parents D Parents-in-law or Grandparents

E Grandparents F Daughter-in-law G Grandchildren H Brothers and sisters

I Live alone J Other _____________

1. Your employment before retirement: ( )

A Not officially employed B Work in government agencies

C Work in public institutions D Work in state-owned enterprises

E Work in a collective enterprise F Work in a private enterprise

G Other (please specify)_____________

1. What is the main source of your monthly income after retirement? (Multiple choices, limited to 3 items) ( )

A Labor income B Retirement pension pension C Unemployment insurance payment

D Minimum living allowance E Property income F Other family members support G Other (please specify)_____________

1. What is your monthly retirement (pension) pension? If you are still working, what is your total monthly income? Choice: pension ( ) pension + work income ( ) work income ( )

A 600 yuan and below B 600-1000 yuan C 1000-2000 yuan D 2000-3000 yuan

E 3000-4000 yuan F 4000-6000 yuan G 6000-8000 yuan H 8000-10,000 yuan

I over 10,000 yuan J Other (please specify)_____________

1. What is your family's monthly income? ( )

A 600 yuan and below B 600-1000 yuan C 1000-2000 yuan D 2000-3000 yuan

E 3000-4000 yuan F 4000-6000 yuan G 6000-8000 yuan H 8000-10,000 yuan

I over 10,000 yuan J Other (please specify)_____________

**2.State of health**

1. Can you take care of your current living situation? ( )

A Completely self-care B Self-care C Completely unable to self-care

1. What do you think of your physical health? ( )

A Very healthy B Relatively healthy C Basic healthy D Relatively unhealthy

E Very unhealthy

1. Are you suffering from chronic diseases? (Common chronic diseases are mainly cardiovascular and cerebrovascular diseases, cancer, diabetes, chronic respiratory diseases, and the central cerebrovascular diseases include hypertension, stroke and coronary heart disease) ( )

A No → Answer 13 B Yes → Answer 12.1 and 12.2

12.1 What chronic diseases do you have? (Multiple choice) ( )

A Hypertension B Stroke C Coronary heart disease D Cancer E Diabetes

F Chronic respiratory disease G Other (please specify)_____________

12.2 Which of the above chronic diseases do you suffer from, which is the longest? How long is it?_____________

1. Have you been to the hospital for medical reasons in the last year?

A hasn't been B probably ______ times

1. Do you check your body regularly? ( )

A Never check → Answer 16 B Occasional check → Answer 14.1

C Periodic check → Answer 14.1 and 14.2

14.1 When was the last time you checked your body? ( )

A within one month B within three months C within six months D within one year

E within one year and above

14.2 How often do you go for regular inspections? ( )

A within one month B within three months C within six months D within one year

E within one year and above

1. The payment method of your medical examination fee is: ( )

A Paid by the company B Paid by the user C Paid by the government D Paid by the child E Other (please specify)_____________

1. In addition to general examination (blood routine and erythrocyte sedimentation rate, urine routine, electrocardiogram, lung function, ophthalmology, chest X-ray, B-ultrasound, etc.), does your physical examination include some special items? ( )

A No, only general inspection B Yes, there are special items, such as: _____________

**3.Elderly medical preferences and expectations**

1. If you feel unwell (except serious illness), what measures will you take? ( )

A goes to the hospital immediately B buys medicine at the pharmacy

C Endurance, the reason is: _____________

D Other measures: _____________

1. If you have a general disease, what level of hospital would you choose to seek medical treatment? ( )

A General Hospital(Tertiary Hospitals) B District Central Hospital (Secondary Hospitals) C Regional Hospital (Secondary Hospitals) D Special Hospital

E Community Clinic(Primary Hospital)

1. Which level of hospital do you think is better for serious diseases? ( )

A General Hospital(Tertiary Hospitals) B District Central Hospital (Secondary Hospitals) C Regional Hospital (Secondary Hospitals) D Special Hospital

E Community Clinic(Primary Hospital)

**Major diseases generally include:** malignant tumors, severe cardiovascular and cerebrovascular diseases, acute myocardial choking, stroke sequelae, major organ transplantation or hematopoietic stem cell transplantation, coronary artery bypass grafting (or coronary artery bypass grafting), chronic liver function Failure and decompensation period, injuries that may cause life-long disabilities, advanced chronic diseases, deep coma, permanent paralysis, severe brain injury, severe Parkinson's disease and severe mental illness.

★**NOTE：According to the Shanghai Municipal Medical Insurance Reimbursement Policy, ①dialysis treatment of severe uremia; ②anti-rejection treatment of kidney transplantation; ③malignant tumor treatment (chemotherapy, endocrine specific treatment, radiotherapy, isotope treatment, interventional treatment, traditional Chinese medicine treatment); ④ Some psychiatric treatments (schizophrenia, moderate to severe depression, mania, obsessive-compulsive disorder, mental retardation associated with mental disorders, epilepsy associated with mental disorders, paranoid psychosis) are considered as four major diseases.**

1. What level of hospital should you go to for follow-up treatment of major diseases? ( )

A General Hospital(Tertiary Hospitals) B District Central Hospital (Secondary Hospitals) C Regional Hospital (Secondary Hospitals) D Special Hospital

E Community Clinic(Primary Hospital)

1. Will you go to a private (private) hospital for treatment? ( )

A Yes, the reasons are: ___________, ___________, ___________.

B No, the reasons are: __________, __________, ___________.

1. If you are sick and need medical treatment, please write down the first three factors that affect you in order from the largest to the smallest? ( )

A Lower fees

B doctor level is higher

C convenient registration

D High-grade, well-equipped hospitals

E Convenient transportation

F Medical reimbursement system

G Hospitals where child care is more convenient

H Hospitals with familiar doctors, relatives and friends

I Doctors and nurses have good service attitude

J Other: __________________

1. What do you think is the most needed health care service organization (person)? According to your needs, sort the importance, 1 is the most needed service organization (person), and so on by analogy. The larger the number, the less the service organization (person);

A Community Clinic (Medical Rehabilitation) ( )

B Family health doctor ( )

C Special Needs Clinic for the Elderly in Hospital ( )

D Public health care service for the elderly ( )

E Sanatorium ( )

F Free medical consultation agency ( )

G Others (please specify): __________________ (　)

1. What do you think is the most needed health care service item? According to your needs, sort the importance, 1 is the most needed service items, and so on by analogy, the larger the number, the less needed service items;

A Regular physical examination ( )

B Regular free medical consultation ( )

C Family Medical ( )

D Home nursing ( )

E Membership Medical Special Needs Service (Optional) ( )

F Low-cost medicines needed to treat chronic diseases ( )

G Others (please specify): _____________________（　）

1. If you suddenly feel unwell, what do you think is the most convenient and possible help: ( )

A Family B Hospital (Hotline 120) C Community Clinic

D Neighbor E Neighborhood Committee F Others (please specify): _____________

1. What do you expect the government to do in health care for the elderly?

**4.Hospital and medical service satisfaction**

1. Do you think the nearest hospital to your home is _______________.

The distance from your home to the nearest hospital is about _________ kilometers.

1. If you go to the hospital, what kind of transportation do you usually take? ()

A Bus B Taxi C Car D Electric car, tricycle or bicycle

E Other (please specify) :_______________

28.1How long does it take for this mode of transportation to the hospital? _______.

1. Which hospital have you been to the most? _______________.
2. Do you often see a specialist outpatient number or a general outpatient clinic? ( )

A Expert Clinic B General Clinic

1. Have you been hospitalized in the last 2 years? ( )

A Yes → Answer 31.1 B No → Answer 32

31.1What is your ward rating? ( )

A Special Needs Ward B General Ward C Other (please specify):_______________

31.2 If it is a special ward, the reason is: ( )

A Economically affordable B. Bed tension in the general ward C. Child arrangement

1. In the past two years (December 2015 to December 2017), your medical expenses totaled ___________ yuan;

After the total cost is deducted from the medical insurance, your personal medical expenses (paid) are: ____________ yuan.

1. Your medical expenses in the last month totaled _______________ yuan;

After the total cost is deducted from the medical insurance, your personal medical expenses (paid) are: ____________ yuan.

1. At present, has your medical expenses affected the normal life of your family? ( )

A Yes B No

1. If medical expenses are reduced, will you go to treat the disease more often? ( )

A will B will not C uncertain

1. Does your neighborhood committee have a community clinic (Health Room) and other medical service organizations? ( )

A No B Yes

36.1 If there is a community health service center (health room), do you think it will help you? ()

A No B Yes

1. Do you think it is convenient to go to the hospital for treatment, registration, etc. ()

A convenient

B Inconvenient, the main problem is: __________________.

C Uncertain

1. Have you ever had a medical dispute with the hospital? ()

A No B Yes, the reason is: _________________.

1. Please make an evaluation of a hospital you visit frequently, and rate the following questions in sequence (5=very satisfied, 4=satisfied, 3=normal, 2=not satisfied, 1=very dissatisfied, the larger the number, the more you The more satisfied).

A If you do not consider your expenses, are you generally satisfied with this hospital (including service, medical level, equipment, etc.)?

1 2 3 4 5

B Overall, are you satisfied with the charges of this hospital?

1 2 3 4 5

C Do you think the drug fees charged by this hospital are reasonable?

1 2 3 4 5

D Do you think the cost of examination and treatment in this hospital is reasonable?

1 2 3 4 5

E Do you think the procedures of this hospital are convenient?

1 2 3 4 5

F Are you satisfied with the environment and sanitation of this hospital?

1 2 3 4 5

G Are you satisfied with the medical equipment in this hospital?

1 2 3 4 5

H Are you satisfied with the professionalism of the medical staff in this hospital?

1 2 3 4 5

I Are you satisfied with the service attitude of the medical staff in this hospital?

1 2 3 4 5

J If you are free to choose, will you visit this hospital next time?

(5=must go, 4=go, 3=not sure, 2=don’t go, 1=don’t go at all, the higher the number, the more you will go)

1 2 3 4 5

**5.Elderly medical insurance methods and degree of protection**

1. Do you know the current types of medical insurance? ( )

A Fully understand B Understand C Generally D Do not understand

E Completely do not understand

1. Do you understand the current medical insurance reimbursement policy? ( )

A Fully understand B Understand C Generally D Do not understand

E Completely do not understand

★If this question chooses A or B→answer 43; choose C or D or E→answer 42

1. If you fully understand the medical insurance reimbursement policy, do you tend to go to these medical institutions to see a doctor? ( )

A Will definitely B May be C Not clear D Will not E will not

1. Before the medical reform, did you enjoy public or medical insurance? ( )

A Enjoyment, medical treatment at public expense

B Enjoyment, enterprise labor insurance

C No enjoyment, reimbursed by wife or child unit _______%

D No enjoyment, completely self-care

1. Now who pays for your outpatient medical expenses? What is the proportion of each? ( )

A All reimbursed by the social medical insurance fund (medical insurance card)

B Excess reimbursement, that is, there is a threshold, the threshold is: _________yuan

C Limit reimbursement, that is, the highest reimbursement amount, the maximum reimbursement limit is: __________ yuan

D Proportion reimbursement, the proportion of government commitment is: ___%; the proportion of own commitment is%

E According to the type of disease, minor illnesses pay for themselves, and major illnesses co-ordinate

F All at your own expense

G Other: _________________

1. According to the current medical system, if you are hospitalized, who will pay for the medical expenses, and what proportion will each bear? ( )

A All reimbursed by the social medical insurance fund (medical insurance card)

B Excess reimbursement, that is, there is a threshold, the threshold is: __________ yuan

C Limit reimbursement, that is, the highest reimbursement amount, the maximum reimbursement limit is: __________ yuan

D Proportion reimbursement, the proportion of each commitment is: ________%

E All at your own expense

F Other: _________________

1. Who bears the self-financed part? ( )

A Borne by the individual B Fully borne by the children

C Individual commitment and child allowance

D Other (please specify): _________________

1. Do you think the biggest problems in the current medical system and policies are: ( )

A Increase in medical expenses, the burden of personal medical expenses is too heavy

B Did not spread medical services to the community

C The interests of ordinary people are not considered much

D The problem of difficulty in seeing a doctor in the hospital is still unresolved

E Others: _________________

1. Have you purchased commercial medical insurance? ( )

A Major illness insurance B General health insurance C Not purchased D Other

1. What are the reasons for buying commercial medical insurance? ( )

A Insurance for children

B Make up for the lack of social insurance reimbursement

C There is no social pooling medical insurance protection

D In order to enjoy good medical conditions during hospitalization

1. In the past two years, have you applied for compensation from an insurance company? ( )

A Yes B No → End questionnaire

1. What is the result of applying for claims? ( )

A Filed for claim, but was not recognized

B. Successful settlement of claims, the proportion of claims ______%

**【End of questionnaire】**

　　【**Description of content**】

1. Outpatient (including emergency) co-ordination fund expenditure: refers to the compensation expenditure paid by the co-ordination fund to the participants' outpatient clinic accumulatively that meets the basic diagnosis and treatment items and the basic medicine catalogue range below 5000 yuan.
2. Inpatient pooling fund expenditure: refers to the expenses paid by the pooling fund for the hospitalization of participants who meet the requirements of the basic diagnosis and treatment items and the scope of the basic medicine catalog, or the compensation for the medicine catalogue of outpatients with serious diseases of more than 5,000 yuan.
3. Major disease burden reduction subsidy (secondary compensation) overall fund expenditure: refers to the use of the overall fund to enjoy in-patient overall compensation for outpatients or 5,000 yuan or more out-patient critical illness overall compensation, whose one-time self-financed medical expenses are still more than 50% of the annual family income expenditure. The compensation standard is the same as that for hospitalization, and it can be increased by 10-20 percentage points for low-income households, five-guarantee households and households with disabilities confirmed by civil affairs.

　　D.What are not covered by Shanghai Medical Insurance

1. What should be paid from the work injury insurance fund;

2. Should be borne by a third party;

3. Should be borne by public health;

4. Seek medical treatment abroad.
